# Mosquitoes in urban green spaces and cemeteries in northern Spain

**DOI:** 10.1186/s13071-024-06263-z

**Published:** 2024-04-02

**Authors:** Fátima Goiri, Mikel A. González, Aitor Cevidanes, Jesús F. Barandika, Ana L. García-Peréz

**Affiliations:** 1 Animal Health Department, NEIKER-Basque Institute for Agricultural Research and Development, Basque Research and Technology Alliance (BRTA), Derio, Spain; 2 Doñana Biological Station, Spanish National Research Council (EBD-CSIC), Seville, Spain; 3 CIBER de Epidemiología y Salud Pública (CIBER ESP), Madrid, Spain

**Keywords:** Culicidae, *Culex pipiens* ecoforms, Diversity, Dynamic populations, Urban green areas, Cemeteries, Blood meal analysis

## Abstract

**Background:**

Mosquitoes inhabiting urban green spaces and cemeteries in Europe represent a crucial facet of public health concern and contribute to the ecological balance. As urbanization intensifies, these areas increasingly serve as vital habitats for various mosquito species, fostering breeding grounds and increasing the risk of disease transmission.

**Methods:**

A study was conducted in the three main cities (inland, coastal, and estuarine) of the Basque Country, northern Spain, to investigate the species composition, abundance, dynamic populations, larval habitats, and host preferences of mosquitoes in urban green spaces and cemeteries. CDC traps and dipping were used to collect mosquitoes for 2 years (2019–2020).

**Results:**

A total of 21 mosquito species were identified, with *Culex pipiens* s.l. being the most abundant and widespread. The three ecological forms of *Cx. pipiens* were found, and *Cx. pipiens pipiens* was the most common in both green areas and cemeteries. Morphological identification together with molecular tools identified 65 COI sequences with high homology. The highest species richness was found in the inland city, followed by the coastal city and the estuarine city. Mosquito abundance was significantly higher in green areas compared to cemeteries and in the coastal and estuarine cities compared to the inland city. The investigation of larval breeding sites highlighted the dominance of *Cx. pipiens* s.l., particularly in semi-artificial ponds, diverse water-holding containers (tyres and buckets) and drainage systems in green areas; in cemeteries, most of the larvae were found in flowerpots and funerary urns. Seasonal activity exhibited variable peaks in mosquito abundance in the different cities, with a notable increase in July or August. Additionally, blood meal analysis revealed that *Cx. pipiens* s.l. fed on several common urban avian species.

**Conclusions:**

Studies on mosquitoes are essential to understand their role in disease transmission and to design targeted and sustainable management strategies to mitigate the associated risks.

**Graphical Abstract:**

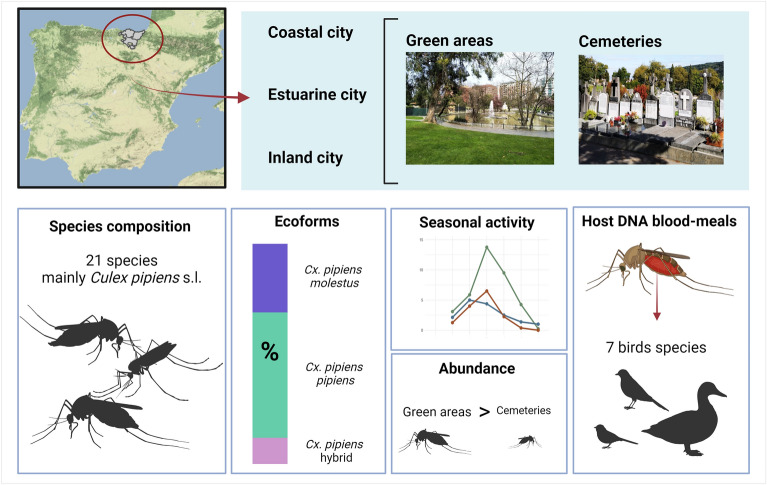

**Supplementary Information:**

The online version contains supplementary material available at 10.1186/s13071-024-06263-z.

## Background

In recent decades, urbanization and landscape anthropization have had an impact on the mosquito community composition and abundance worldwide [1]. Thus, urbanization has been recognized as a major driver of biodiversity change, often resulting in a decrease in the number of species in urban environments. This loss in mosquito biodiversity in urban areas is a consequence of the simplification of the habitat structures and alteration of trophic interactions [2]. However, urbanization increases the availability of human-made water habitats, providing more suitable breeding sites for some native and invasive mosquito species in various types of artificial containers [3]. Invasive species like *Aedes aegypti* in the US [4] or *Aedes albopictus* in Europe have colonized and thrived in urban environments [5]. Consequently, these changes have led to an increase in the global incidence of mosquito-borne diseases, especially in Europe [6]. Urban green areas are the locations in cities where the most vegetation cover can be found. Usually, these areas are destined and designed for the citizens to engage with nature, enjoy leisure time, and perform physical activities [7, 8]. However, these environments harbour the ideal conditions for mosquito proliferation [8–10]. Besides, numbers of predators of mosquitoes, such as fish, amphibians, or aquatic invertebrates, are usually reduced in these urban environments [11]. Additionally, these green ecosystems provide a wide range of animal and human hosts to feed on [12]. Therefore, these spaces could serve as hotspots for the proliferation of some mosquito species. A characteristic of urban spaces is the high availability of wastewater drainage systems and artificial containers, which when filled with water become habitats for immature stages of mosquitoes [13]. Also, urban cemeteries are considered suitable habitats for the proliferation of both native and invasive mosquito species [14, 15]. These types of environments provide shelter habitats such as bushes and trees for adult mosquitoes as well as immature stage habitats like flowerpots [13, 14].

Usually, urban areas are generally warmer than periurban and rural areas because of the urban ‘heat island effect’ [16]. This effect is caused by the lack of vegetation cover and the presence of cemented areas in these environments [17]. The urban heat island effect shortens the life cycle of mosquitoes, consequently increasing their abundance [18]. Among Culicidae, *Culex pipiens* s.l. is widely distributed throughout Europe and is the most common species in urban areas [19, 20]. Besides, species like *Ae. albopictus*, with an important role in different arbovirus transmissions, has a higher presence in these human-modified landscapes [12]. This is due to their adaptation and capacity for breeding and developing in artificial and human-made containers (e.g., road drains, flowerpots, cans, or buckets), which not all species are able to use [21, 22]. Cemeteries are considered adequate hotspots for these invasive mosquitoes, leading to surveillance efforts in these settings [15, 23, 24]. Therefore, with cities continuing to expand, it is imperative to monitor the distribution and abundance of mosquito species in green and grey areas. This is crucial for their management and control [25] to prevent mosquito-borne diseases.

The aim of the study was to assess the diversity, abundance, seasonal dynamics, larval habitats, and trophic preferences of mosquitoes in urban green areas and urban cemeteries from the three main cities of the Basque Country (northern Spain). A morphological and molecular approach was performed to identify mosquito species, their ecoforms, and/or sibling species. We also evaluated the factors associated with their abundance, species richness, and potential larval sites.

## Methods

### Study area

**Fig. 1:**
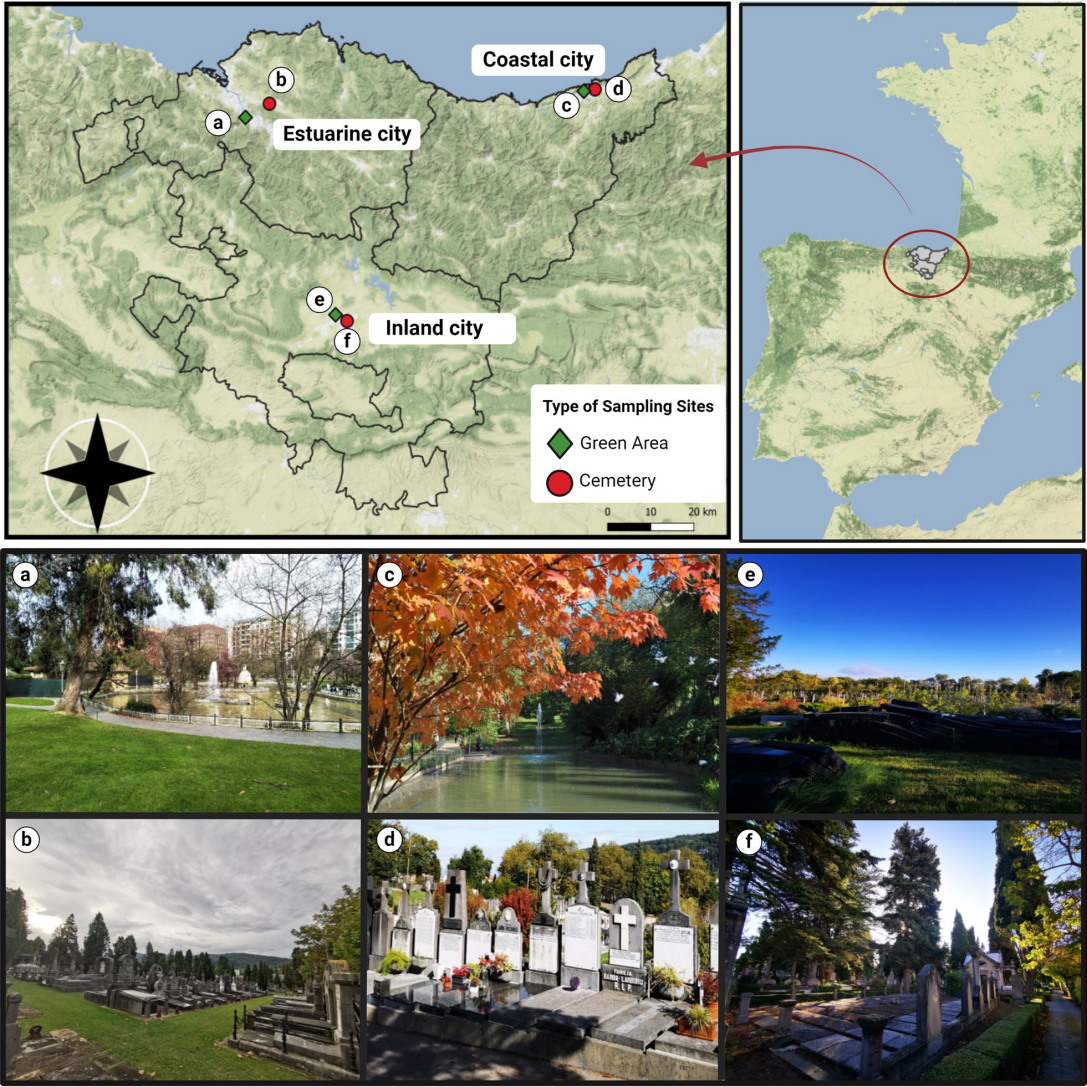
Map of the Basque Country (northern Spain) indicating the location of the three cities and the six sampling sites: Estuarine city (**a** Casilda park; **b** cemetery); coastal city (**c** Kristinaenea park; **d** cemetery); inland city (**e** municipal plant nursery; **f** cemetery). [**a**, **c**, **e**: green urban areas (green diamonds); **b**, **d**, **f**: cemeteries (red circles)]

The study took place in the three main urban areas of the Basque Country, northern Spain: the inland city of Vitoria-Gasteiz (province of Araba), the coastal city of Donostia-San Sebastián (province of Gipuzkoa), and the estuarine city of Bilbao (province of Bizkaia) (Fig. 1). The inland city, Vitoria-Gasteiz, is the capital of the autonomous region of the Basque Country. It has an extension of ca. 276 km^2^, with around 250,000 inhabitants and a population density of ca. 898 inhabitants/km^2^ [26, 27]. In 2010, the European Union (EU) awarded it the title of 2012 European Green Capital, and in 2019, it was also recognised as a “Global Green City” by the United Nations (UN) due to its environmental policies, green infrastructures, and sustainable mobility [28]. The estuarine city, Bilbao, covers an area of 41.60 km^2^, with ca. 345,000 inhabitants and a population density of ca. 8296 inhabitants/km^2^ [26, 27]. Until the 1980s, it was primarily an industrial area [29]. The coastal city of Donostia-San Sebastián spans ca. 61 km^2^, with ca. 180,000 inhabitants and a population density of around 2980 inhabitants/km^2^ [26, 27]. Both the coastal and estuarine cities enjoy Atlantic climate, characterised by temperate and wet conditions throughout the year. In contrast, the inland city has a transitional climate between Atlantic and Mediterranean, with cold winters and drier, warmer summers [30].

In each city, a green area and a cemetery were selected (Fig. 1). The criteria used to choose these sites was based on easy accessibility, suitability, and lower risk of vandalism. The green areas were in the heart of the selected cities. In the inland city of Vitoria-Gasteiz, the landscaped area is used mainly for gardening, featuring small tree forest patches, greenhouse area, as well as areas with debris and tyres. In the estuarine city of Bilbao, there is an extensive garden with grass, scattered trees, and a large ornamental fountain occupying part of the territory. In the coastal city of Donostia-San Sebastián, there is an extensive, green, leafy, damp, and highly vegetated garden with a wide variety of plant and tree species along with several semi-artificial water pools. A list of the hosts that were most frequently observed during field visits is provided (Additional file 1: Table S1).

The three selected cemeteries were different in terms of structure, vegetation, and mainly the number of water-holding containers, probably reflecting distinctive cultural habits. All cemeteries were soil-cemented and had mixed trees and green patches.

### Field sampling approach

Mosquito trapping was carried out fortnightly during two periods: from 1 May to 31 October 2019 and from 1 June to 30 November 2020. The COVID-19 pandemic in 2020 forced us to start the field sampling 1 month later. Two CDC miniature traps (model 1212, John Hock, Gainesville, FL, USA) were deployed in each sampling site, equipped with incandescent light, and baited with ca. 1.5 kg of dry ice (CO_2_). Traps were placed at least 100 m apart and were placed on two distinctive habitats. They were positioned in shady, humid, and windless areas as these are locations where mosquitoes tend to rest. All traps were set up early in the morning and recovered 24 h later. In addition, in 2019, mosquito larval sites were searched and sampled once per month in a radius of 200 m around CDC traps. In green areas, immature mosquito stages were collected using a dipper (600 ml) as detailed by González et al. [31]. These samples were then transported to the laboratory and kept in mosquito breeders (Bioquip Products, USA) until adult emergence. A total of seven, four, and five types of larval sites were inspected each month in the inland city, estuarine city, and coastal city, respectively. In cemeteries, instead of sampling around a 200 m radius of each CDC trap position, we sampled a maximum surface of 2500 m^2^ per site. The cemeteries of the inland and estuarine cities contained a low number of containers (*n* = 149), whereas the cemetery in the coastal city included > 340 containers (Additional file 2: Table S2). The number of containers inspected, number of containers with water, and number of containers positive for mosquitoes are detailed in Additional file 2: Table S2.

### Mosquito species identification

In the laboratory, mosquitoes were sorted by sex and physiological status (blood-fed, gravid, and unfed). The species identification relied on morphological features of females and male genitalia using taxonomic keys [32, 33]. Examination of genitalia of males allowed for differentiation between *Culex torrentium* and *Cx. pipiens* s.l. Damaged or morphologically indistinguishable mosquito specimens were identified by molecular methods. Briefly, genomic DNA extraction was carried out with NZY Tissue gDNA isolation kit (NZYTech, Lisboa, Portugal) followed by a PCR targeting cytochrome c oxidase I (COI) with primers C1-J-1718 and C1-N-2191 as described by Delgado-Serra et al. [34]. PCR amplicons were then purified using ExoSAP-IT (Applied Biosystems, Thermo Fisher Scientific, Vilnius, Lithuania) and submitted for Sanger sequencing (Eurofins Genomics, Germany). The sequences obtained were analysed using Geneious Prime software (v.2022.2.2) and compared with the GenBank database through nucleotide sequence homology searches on the network server of the National Center for Biotechnology Information (NCBI) using BLAST or at the Barcode of Life Database (BOLD) (http://www.boldsystems.org/index.php).

Mosquitoes belonging to the *Anopheles maculipennis* s.l. complex were identified to the species level using a PCR-RFLP assay targeting polymorphisms in the Internal Transcribed Spacer 2 (ITS-2) [35] with primers described in Collins and Paskewitz [36]. Similarly, a subsample of the specimens morphologically identified as *Cx. pipiens* s.l. of each green area and cemetery were analysed by molecular methods (ca. 25%; *n* = 146; 44 from 2019 and 102 in 2020) to determine their ecoform (*Cx. pipiens pipiens*, *Cx. pipiens molestus*, and its hybrids) by targeting the flanking region of the CQ11 microsatellite [37].

### Host blood meal analysis

Vertebrate host species of blood-fed and gravid females collected in 2019 were investigated at the Centre for Biodiversity Genomics, University of Guelph (Guelph, ON, Canada). Host feeding patterns were identified using a metabarcoding-like approach with next-generation sequencing (NGS) technology as previously described [38, 39]. Identification was considered valid only when the query sequence matched the reference sequence with at least 95% nucleotide identity. Detailed specimen records and sequence information were uploaded to the Barcode of Life Database (BOLD-http://www.boldsystems.org) and can be found within the Working Group 1.4 Initiative “Human Pathogens and Zoonoses” container “MCBCS-Surveillance of mosquitoes and *Culicoides* in the Basque Country, Spain.” The digital object identifier (DOI) for publicly available projects in BOLD is doi:dx.doi.org/10.5883/DS-MQBMBC.

### Data analysis

Statistical analyses were performed using R statistical software version 4.2.0 [40]. Differences between the abundance of the most trapped mosquito species in green areas and cemeteries were analysed using non-parametric Wilcoxon rank sum test. Chi-squared test and Fisher exact test were used to evaluate the differences between *Cx. pipiens* ecoforms and the type of sampling area (green area vs. cemetery) and city. Multivariate generalized linear models (GLM) were run to evaluate the differences in the overall abundance of mosquitoes (catches/trap/night), related to sampling site (green urban area, cemetery), city (estuarine, coastal, and inland city), year of sampling (2019, 2020), and month of sampling (June to October, shared period for both periods of sampling). A negative binomial generalized linear model (NBGLMs) was employed [41] because of the data over-dispersion of the mosquito abundance, using the MASS package [42]. Using the “MuMIn” package and “dredge” function [43], the best models were selected based on Akaike information criterion and corrected to sample size (AICc). The overall fit of the model was evaluated with a likelihood ratio test, comparing the best model with the null model. Species richness (S) and Shannon-Wiener diversity (H’) were calculated to compare biodiversity among cities, sampling areas, and type of sampling sites (green area vs. cemetery) using the “diversity” function in the “vegan” package [44].

## Results

### Species composition and abundance

A total of 846 mosquitoes (682 females and 164 males) were collected by CDC suction traps in green areas and cemeteries from the Basque Country (northern Spain). In 2019, a smaller number of mosquitoes (*n* = 263, 207 females and 56 males) were captured compared to the catches in 2020 (*n* = 583 mosquitoes, 475 females and 108 males).

Morphological and molecular analyses allowed for the identification of 21 mosquito species (one invasive and 20 native mosquitoes), encompassing six *Aedes* spp., seven *Culex* spp., four *Anopheles* spp., three *Culiseta* spp., and one *Coquillettidia* species (Table 1). Among them, three species (*Cx. pipiens* s.l., *Culiseta longiareolata*, and *Culex hortensis*) were found in the three cities. Regarding abundance, the highest mean abundance belonged to the green area of the estuarine city (6.16 ± 1.37) and the lowest mean to the cemetery in the same city (1.20 ± 0.26) (Table 1). Overall mean abundance of green areas was higher (4.13 ± 0.55) than in cemeteries (1.51 ± 0.17) (*W* = 8244; *P* < 0.001). Interestingly, a single specimen of *Ae. albopictus* was captured with CDC traps in the cemetery of the estuarine city in 2020.

**Table 1:** Culicidae trapped by baited CDC traps in the six urban environments in the Basque Country (northern Spain) in 2019 and 2020

Culicidae	Green areas			Cemeteries			Total
	Estuarine city	Coastal city	Inland city	Estuarine city	Coastal city	Inland city	
*Aedes albopictus*	0	0	0	1	0	0	1
*Ae. caspius*	1	0	0	0	0	0	1
*Ae. detritus*	0	1	0	0	0	0	1
*Ae. geniculatus*	0	9	1	0	0	0	10
*Ae. rusticus*	0	0	6	0	0	0	6
*Ae. vexans*	0	1	4	0	0	0	5
*Anopheles atroparvus* s.s.	0	0	1	0	0	0	1
*An. claviger*	0	0	12	0	0	1	13
*An. maculipennis* s.s	0	0	2	0	0	0	2
*An. plumbeus*	0	8	0	0	3	0	11
*Culiseta annulata*	0	13	7	0	2	1	23
*Cs. longiareolata*	37	12	12	5	44	8	118
*Cs. subochrea*	0	0	1	0	0	0	1
*Culex hortensis*	0	0	0	3	6	1	10
*Cx. mimeticus*	0	1	0	0	0	0	1
*Cx. modestus*	1	0	0	0	0	0	1
*Cx. pipiens* s.l./*Cx. torrentium*^*a*^	267	144	65	51	48	47	622
*Cx. territans*	0	0	1	0	0	0	1
*Cx. theileri*	0	0	6	0	0	1	7
*Coquillettidia buxtoni*	0	5	0	1	0	0	6
Unidentified species	2	0	0	0	2	1	5
Total	308	194	118	61	105	60	846
Mean ± SE^b^	6.16 ± 1.37	3.88 ± 0.53	2.36 ± 0.66	1.22 ± 0.29	2.10 ± 0.33	1.20 ± 0.26	3.32 ± 0.39

^a^At least five specimens of *Cx. torrentium* were identified by morphological (1 male in the cemetery of the coastal city) or molecular (3 females in the green area of the coastal city and 1 female in the cemetery of the estuarine city) methods. Males and females were pooled

^b^*SE* standard error

*Culex pipiens* s.l./*Cx. torrentium* (*n* = 622, 73.5%) was significantly the most abundant species (*W* = 51,945; *P* < 0.001), followed by *Cs. longiareolata* (*n* = 118, 13.9%). In all sampled areas, the most captured species was *Cx. pipiens* s.l./*Cx. torrentium*, being more abundant in green areas (76.7% of the total catches, 476/621) than in cemeteries (64.9% of the total catches, 146/225) (*W* = 8644, *P* < 0.001).

The molecular analysis yielded 65 barcoding COI sequences of 443–525 bp length with 97–100% homology compared with GenBank sequences. A selection of these sequences (*n* = 21 and 13 species) was deposited in GenBank under accession numbers PP218317-PP218337.

**Fig. 2:**
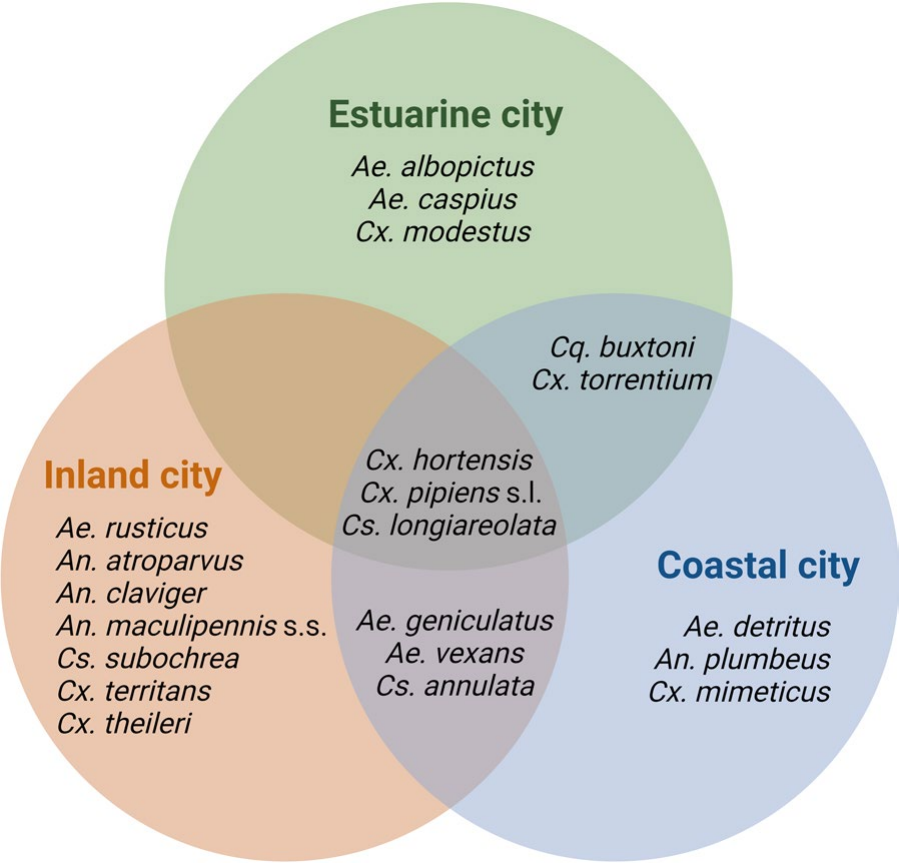
Venn diagram represents the Culicidae species found in urban environments of the three main cities of the Basque Country (northern Spain)

**Fig. 3:**
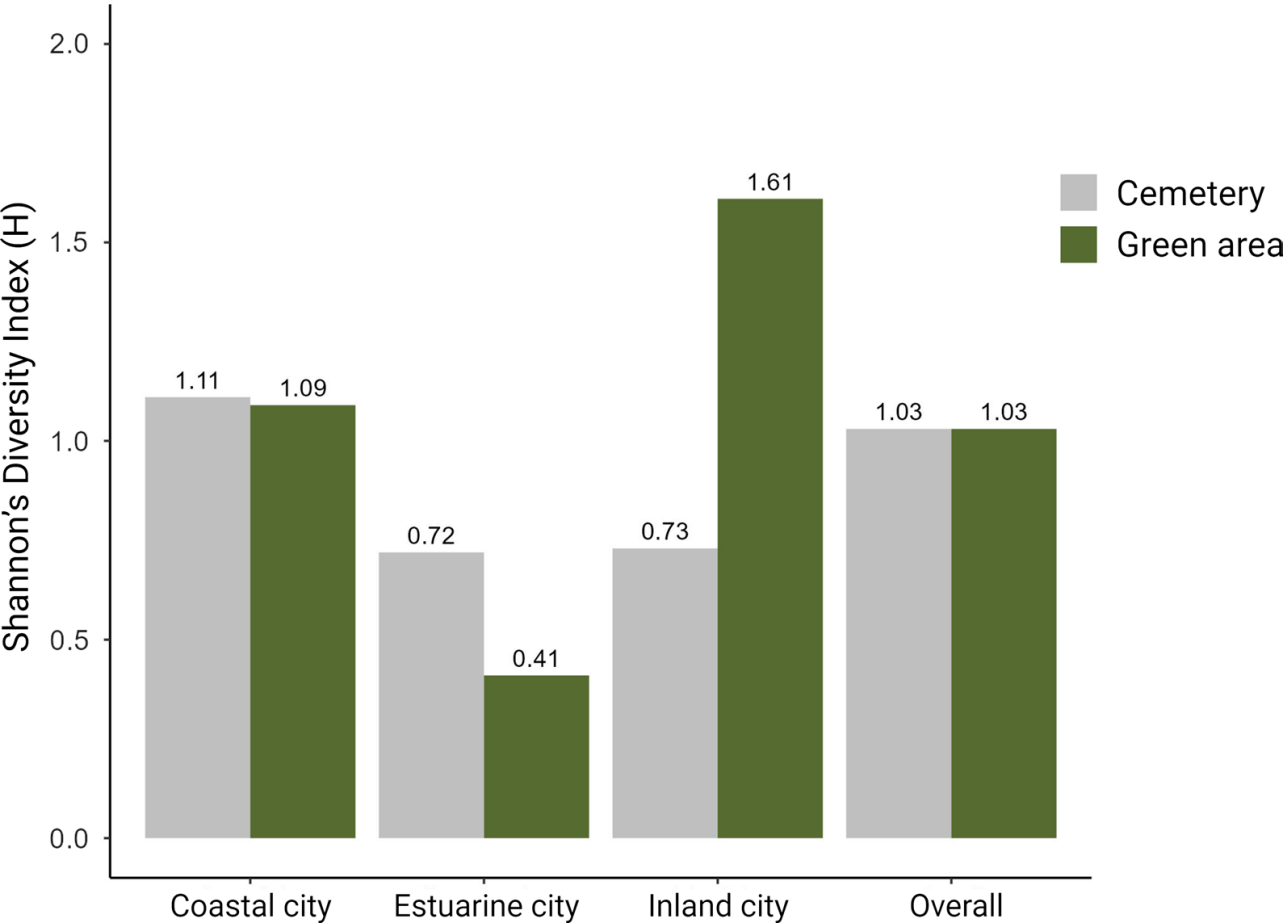
Shannon’s diversity index (H’) by city and type of sampling site

The three specimens initially included as *An. maculipennis* s.l. complex were subsequently identified as *Anopheles atroparvus* (*n* = 1) and *An. maculipennis* s.s. (*n* = 2). The inland city harboured the highest species richness with 13 different species, followed by the coastal city (*n* = 11) and the estuarine city (*n* = 8) (Fig. 2). Shannon’s diversity index (H’) showed a higher diversity in the inland city (*H*’ = 1.39), followed by the coastal city (*H*’ = 1.24) and estuarine city (*H*’ = 0.49). The diversity was identical in both green areas (*H*’ = 1.03) and cemeteries (*H*’ = 1.03). The highest diversity index was found in the green area of the inland city (*H*’ = 1.61) (Fig. 3).

**Fig. 4:**
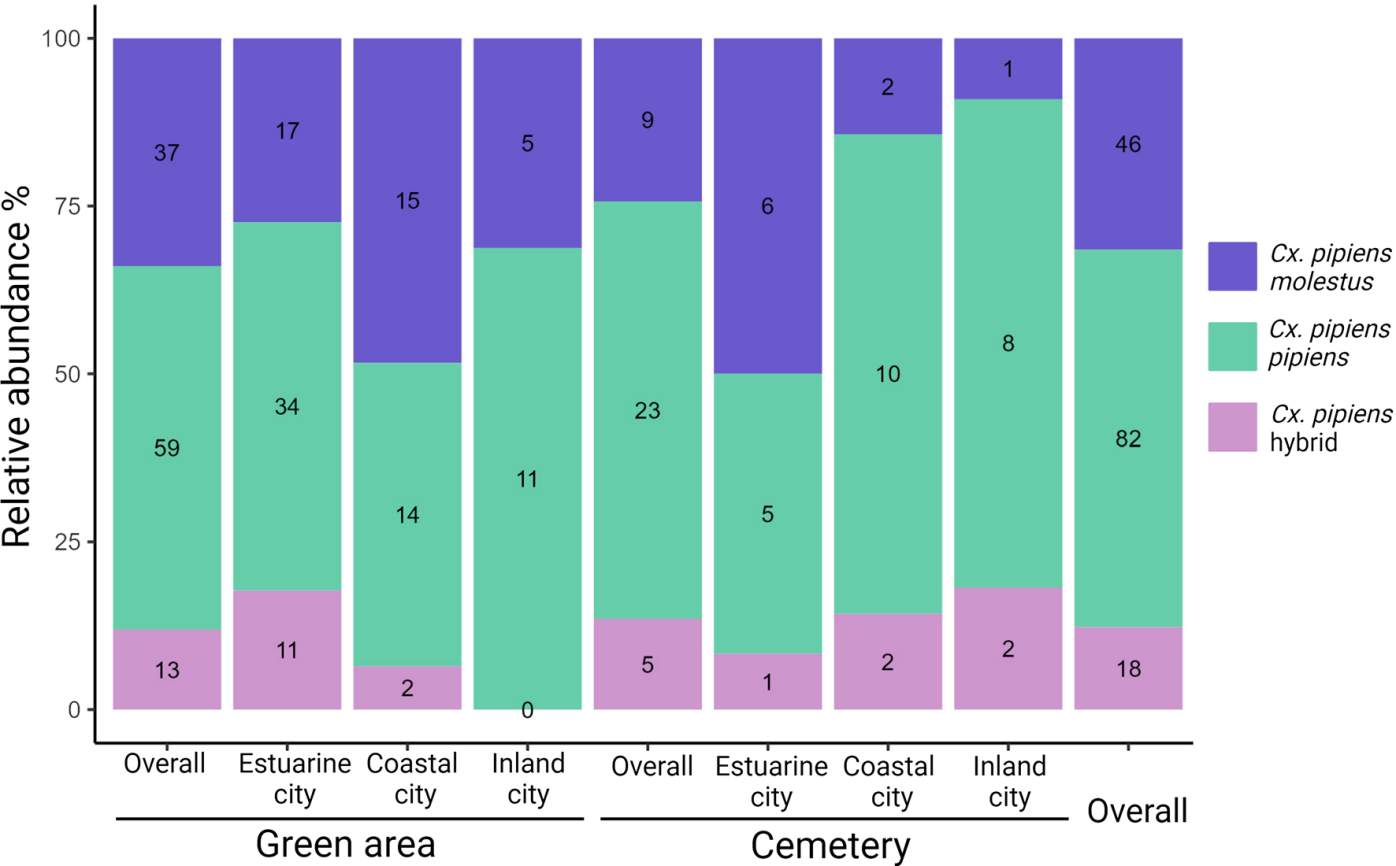
Relative abundance of *Culex pipiens* s.l. ecoforms in the six urban environments in the Basque Country (northern Spain) (number inside the bars corresponds to absolute numbers)

The approach to determine *Cx. pipiens* ecoforms showed *Cx. pipiens pipiens* (*n* = 82, 56.2%) as the most abundant, followed by the *Cx. pipiens molestus* (*n* = 46, 31.5%) and hybrid form (*n* = 18, 12.3%) (Fig. 4). Overall, no significant differences were found between green area and cemetery in the distribution of the three ecoforms (*χ*^2^ = 1.185, *P* = 0.553). No significant differences were found among the three cities (*χ*^2^ = 4.366, *P* = 0.359), the ecoform *pipiens* being the most abundant in all the cities, followed by the form *molestus* (Fig. 4).

### Seasonal activity

**Fig. 5:**
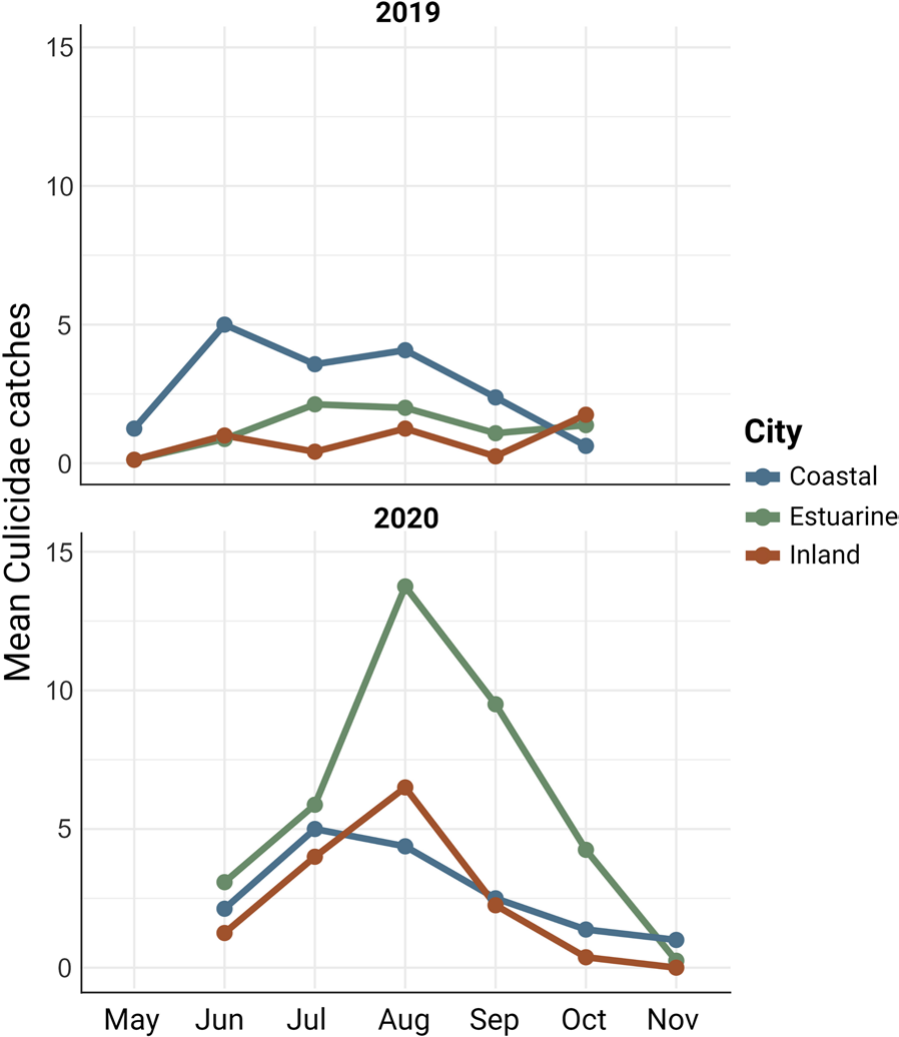
Seasonal flight activity of Culicidae captured in CDC traps at the three cities of northern Spain in 2019 and 2020

Considering the mosquito catches obtained by CDC traps in 2019 and 2020, mosquito flight activity extended throughout the entire sampling period. Population dynamics during 2019 in the three cities showed a moderate increase during the first months of sampling, with peaks in June and August in the coastal city, in July and August in the estuarine city, and June, August, and October in the inland (Fig. 5). In 2020, the estuarine city showed the highest mosquito abundance. All the cities experienced an increase in mosquito abundance during the initial months of sampling, followed by a progressive decline from August onwards (Fig. 5).

### Larval rearing sites

Eight different mosquito species were identified in larval habitats, including *Cx. pipiens*, *Cx. torrentium*, *Cx. hortensis*, *Culex territans*, *Anopheles claviger*, *Culiseta annulata*, *Cs. longiareolata*, and *Aedes geniculatus*. Most larvae were collected from green areas (*n* = 319) compared with cemeteries (*n* = 143) (Table 2). Overall, *Cx. pipiens* s.l. (*n* = 340, 73.6%) was the most abundant species in the larval sites from all the sampling areas, followed by *Cs. longiareolata* (*n* = 69, 15.0%) (Table 2). The most prolific mosquito larval sites in green areas were pools of water (semi-artificial ponds), diverse water-holding containers (tyres and buckets), and drainage systems, while in cemeteries flowerpots supported most of the larval abundance and pots and funeral urns to a lesser extent. Larvae were found in all types of water-holding materials in cemeteries (plastic, ceramic, metal, and/or marble). *Culex pipiens* s.l. and *Culiseta* spp. bred in a wide variety of artificial and natural water-holding containers, whereas *Ae. geniculatus* preferred to rear exclusively on tree holes together with *Cx. pipiens*, *Cx. territans*, and *Cx. torrentium*, albeit to a lesser extent. The latter was found cohabiting in the same habitats as *Cx. pipiens*. Interestingly, a single *An. claviger* was found breeding in a large plastic tray. It is interesting to note that artificial urban lakes might contain larvae of *Cx. pipiens* on the muddy and shady edges. Huge differences were recorded in the mosquito abundance among the three study sites, which was a reflection of the number of available larval sites, i.e. in the green area of the estuarine city and its cemetery few developmental sites were found.

**Table 2:** Culicidae species found in larval habitats in the six urban environments studied in the Basque Country (northern Spain) during 2019

Culicidae species	Green areas				Cemeteries				Total
	Estuarine city	Coastal city	Inland city	Total	Estuarine city	Coastal city	Inland city	Total	
*Culiseta annulata*		7	1	8					8
*Cs. longiareolata*		3	28	31		38		38	69
*Culex pipiens* s.l.*/Cx. torrentium*	21	114	118	253	30	57		87	340
*Cx. hortensis*						18		18	18
*Cx. territans*		19		19					19
*Anopheles claviger*			1	1					1
*Aedes geniculatus*		7		7					7
Total	21	150	148	319	30	113	0	143	462

### Analyses of variables affecting mosquito abundance

The negative binomial model showed a positive association between the total abundance of mosquitoes with the type of sampling area, being significantly higher in the green areas compared to the cemeteries (Table 3). Moreover, the abundance of mosquitoes collected in CDC traps was also positively associated with the city, the year, and the month of sampling, the abundance being significantly higher in coastal and estuarine cities, in the year 2020, and in the months of July and August (Table 3).

**Table 3:** Summary of the best negative binomial regression model for total number of Culicidae per CDC trap and night

Variables	Abundance per CDC trap/night		
	Est ± SE^a^	*z*^b^	*P*-value^c^
Type of sampling area			
Cemetery	Ref.^d^		
Green area	0.90 ± 0.16	5.66	< 0.001
City			
Inland	Ref.		
Coastal	0.66 ± 0.20	3.36	< 0.001
Estuarine	0.59 ± 0.20	2.94	0.003
Year of sampling			
2019	Ref.		
2020	0.74 ± 0.16	4.64	< 0.001
Month of sampling			
October	Ref.		
June	0.32 ± 0.26	1.20	0.229
July	0.63 ± 0.25	2.49	0.013
August	0.98 ± 0.25	3.84	< 0.001
September	0.31 ± 0.27	1.21	0.227

^a^Est ± SE = estimate ± standard error

^b^*z* = statistic *z*-value

^c^*P* = *P*-value

^d^Ref. = reference category

### Host DNA blood meals

Few blood-fed/gravid specimens were captured (*n* = 17). Host DNA amplification success in mosquito samples was 47.1% (8/17). Female mosquitoes that failed in identifying host DNA were categorised within advanced Sella stages. *Culex pipiens* s.l. fed on seven species of urban birds (Table 4). It was not possible to identify the host DNA blood meal in five *Culiseta* spp.

**Table 4:** Blood meal host identification in Culicidae in the six urban environments studied in the Basque Country (northern Spain) during 2019

City	Culicidae		
	No.^a^	Species	Host DNA (no.^b^)
Estuarine city	3	*Culex pipiens* s.l	*Turdus merula* (1)
Coastal city	1	*Culiseta annulata*	–
	4	*Cs. longiareolata*	–
	9	*Cx. pipiens* s.l	*Turdus merula* (1) *Turdus philomelos* (1) *Anas platyrhynchos* (1) *Erithacus rubecula* (1) *Passer domesticus* (1) *Serinus serinus* (1) *Serinus canarius* (1)

^a^Total number of blood-fed or gravid mosquito females analysed

^b^In parentheses: the number of mosquitoes in which host DNA was identified

## Discussion

Globalization and landscape anthropization affect the composition, distribution, and abundance of mosquito communities in urban areas, thereby impacting the incidence of mosquito-borne diseases [12, 45]. Mosquito abundance is linked to landscape composition in urban green spaces [2, 3, 10, 46]. For example, in some locations, an increased presence of water and aquatic plants is correlated with higher mosquito density, while greater coverage of woodland plants appears to reduce mosquito abundance [10]. However, other studies showed that the distribution of mosquitoes depends on the life stage and influence of microclimatic conditions [47]. For this reason, we aimed to extend the study of Culicidae in northern Spain to determine which species inhabit urban areas and assess their potential risk to public health. The number of mosquito species and their abundance was slightly lower in the current study compared to pristine habitats in the same region [48]. Therefore, in none of the three cities did health authorities consider the application of adulticidal or larvicidal treatments necessary. Overall, mean abundance per trap and night was low and similar to findings reported in urban areas from other Spanish regions [3, 49], with *Cx. pipiens* being the most abundant species.* Culex pipiens* is the most common and predominant species in urban areas in both Spain and the northern hemisphere, and it serves as a vector of several pathogens such as West Nile virus (WNV) [50] and Usutu virus [51]. In our study, this species was found breeding in all kind of habitats (15/16 types), whether natural or artificial nature. This species has two ecologically distinct ecoforms and a hybrid; the *molestus* form is usually described as the below-ground form, mammophilic, and capable of laying eggs without a blood meal [32], while the *pipiens* form is an ornithophilic above-ground form [52]. This ecoform categorization is well defined in northern parts of Europe, but in southern Europe, this distinction is not as clear [52]. In our study, like in other studies carried out in urban areas of the Iberian Peninsula [49, 53], *Cx. pipiens pipiens* was the most abundant form followed by *Cx. pipiens molestus*. However, in some other Spanish regions, *Cx. pipiens pipiens* are more frequently found in natural areas [54]. Interestingly, our study showed no differences in the frequency of these forms between green areas and cemeteries. Regarding hybrids, the percentage found in this study (12.3%) is lower compared to levels observed in other Spanish regions [49]. These differences might be attributed to host availability and habitat conditions, which might favour hybridization.

Along with *Cx. pipiens* s.l., *Cs. longiareolata* is considered the second most abundant and widely distributed species in many regions of Spain [55]. This mosquito species is commonly found breeding in artificial containers in urban environments [56]. In our study, this species was recorded developing in artificial habitats (track tyres, buckets, flowerpots, funeral urns, small pools of water, and sewer systems) in both green areas and cemeteries. *Culiseta longiareolata* is considered ornithophilic [32] but also feeds on mammals, including humans [57]. Although it has been considered of low interest for public health, this species could potentially act as vector of avian pathogens [33].

Our study also showed that the highest mosquito abundance was recorded in the green urban areas, especially in 2020, when the COVID-19 pandemic took place. This difference could be explained by climatic variables (not recorded in this study) or by the management of green areas, which, due to the pandemia, were neglected and presumably more resting sites and hiding places were available compared to the previous year. The green areas investigated in this study showed a higher diversity of mosquitoes than the cemeteries. Most of the species found in green areas and cemeteries have been reported in previous studies conducted in the territory. *Aedes geniculatus*, however, has only been previously identified through egg analysis by molecular tools in *Aedes* surveillance programs [58]. This mosquito species exhibited aggressive biting behaviour towards humans during the field work. Small size water-filled holes in alder trees (*Alnus* sp.) were used by this species as developmental sites, in line with other studies [59]. Notably, most of the species found in our study have also been recorded in nearby naturalised areas [48]. This is the case of *Aedes caspius*, *Aedes detritus*, and* Culex modestus*, which usually breed in brackish and saline water [60–62], that have been found in estuarine and coastal cities. Only five specimens of *Ae. rusticus* were found in the green area from the inland city, which suffers more extreme climatic conditions than the estuarine and coastal cities, both located near the coast and influenced by a mild Atlantic climate. This could explain the limits of the distribution of *Ae. rusticus*.

Cemeteries are widely recognized as ideal locations for mosquito proliferation as they provide multiple habitats for their development [24]. Urban cemeteries are also very accessible and frequently visited, providing blood sources from either visitors or on-site workers [63]. However, the productivity of cemeteries as reservoirs of immature mosquito larvae depends on many factors, such as cultural practices and religious customs [13]. This explains why the cemetery in the coastal city was very productive in terms of the number of water-filled containers, particularly flowerpots and funeral urns, while the larval sites in the other two cities were scarce or inexistent. This may be due to the different management of the individual cemeteries, where visitors are encouraged to minimize the flowerpots in the graves and monuments. Cemeteries are also routinely surveyed in Europe to monitor invasive *Aedes* species such as *Ae. albopictus*, *Aedes japonicus*, and *Ae. koreicus* [63–65]. In fact, in our study a single specimen of *Ae. albopictus* was captured in one of the three cemeteries investigated, although this invasive species has been present in the Basque Country since 2014 [66]. Although the tiger mosquito is currently widely distributed in the region [58], it seems that the population density at the time of sampling was still not high enough to be collected. It is also interesting to note that a second invasive *Aedes* species (i.e. *Ae. japonicus*) present in the Basque Country [58, 67] was not recorded in the study.

In our study we found that multiple artificial containers in cemeteries might serve as larval sites for mosquito species such as *Cx. pipiens* s.l./*Cx. torrentium*, *Cs. longiareolata*, and *Cx. hortensis*. Interestingly, *Cx. hortensis* was exclusively detected in cemeteries by both CDC traps and larval dipping. Despite limited knowledge about the phenology and general biology of *Cx*. *hortensis* [32], this species is commonly found in Spain, France, Italy, and Greece up to central Europe [68]. In our study, *Cx. pipiens* s.l. was the most abundant species sampled in the immature stage. However, previous studies carried out in the same area indicated that *Cx. hortensis* was the most abundant species found in larval habitats in urban areas [31].

From the 21 species of mosquitoes identified in cemeteries and urban green areas, *Ae. albopictus* has a real impact on human health [69]. *Culex pipiens* s.l.*, Cx. modestus*, and *Ae. detritus* have also been confirmed to be vectors of WNV in Europe [70–72], with *Cx. pipiens* s.l. being one of the most important vector species of this pathogen [73, 74]. Other mosquito species such as the ornithophilic *Aedes vexans*, which primarily feed on birds, have been found to be a competent vector in the transmission of WNV, acting as a bridge vector between birds and humans [75, 76]. *Anopheles plumbeus* has gained interest in Europe as it has been identified as a potential malaria vector [77–79]. Besides, among the members of the *An. maculipennis* complex, it is relevant to note the detection of *An. atroparvus* in the green area of the inland city, as it is a recognised historic vector of malaria [77].

Blood meal analysis is a fundamental tool for understanding the ecology of mosquitoes [80], as their feeding habits are critical factors in the transmission of vector-borne pathogens [81]. Owing to the low number of blood-fed specimens recorded in our study, it is difficult to establish robust conclusions. Nonetheless, the trophic habits of *Cx. pipiens* s.l. showed a pronounced preference for avian hosts, in line with previous works performed in urbanised habitats [39], even when other mammal hosts, such as pedestrians and dogs, may coincide with the peak time of mosquito activity. However, in the metropolitan area of Barcelona, *Cx. pipiens* showed preference for birds but also for humans, dogs, and cats [82]. Host choice is host dependent as seen in the urban zoos of Barcelona where *Cx. pipiens* also showed mixed feeding habits [83].

## Conclusions

This study provides new insights into the abundance and mosquito community composition in green urban areas and cemeteries of northern Spain. Despite the relatively low abundance of mosquitoes in urbanized areas, the most trapped species are regarded as crucial vectors for various pathogens. Therefore, health authorities should adopt a multi-faceted approach to mosquito management, including the implementation of biological treatments in mosquitoes breeding sites and the removal of water-filled containers. Other effective strategies may include community engagement and education programs to raise awareness about reducing stagnant water areas in residential and public spaces. Regular inspection and maintenance of drainage systems to prevent water accumulation, as well as the use of environmentally friendly larvicides to target mosquito breeding grounds could further bolster control measures. Collaborative efforts among health departments, local authorities, and community participation can enhance the efficacy of control programs aimed at reducing mosquito populations and the risk of associated disease transmission. Besides, the current results indicate that *Cx. pipiens* s.l. is the most common taxon of the Culicidae family in urban areas, exhibiting an ornithophilic feeding preference. A better understanding of the trophic behaviour/preferences of these Diptera pests can contribute to understanding the transmission patterns of pathogens of public health interest.

### Supplementary Information

**Additional file 1: Table S1.** Commonly encountered potential hosts identified during field visits to green areas.

**Additional file 2: Table S2.** Number of containers inspected, filled with water and with presence of larvae, by city and month.

## Data Availability

All data are included in the article and in the Supplementary information.

## References

[1] Wilke ABB, Chase C, Vasquez C, Carvajal A, Medina J, Petrie WD (2019). Urbanization creates diverse aquatic habitats for immature mosquitoes in urban areas. Sci Rep.

[2] Ferraguti M, Magallanes S, Ibañez-Justicia A, Gutierrez-Lopez R, Logan JG, de la Puente JM (2022). Implication of human landscape transformation on mosquito populations. Ecology of diseases transmitted by mosquitoes to wildlife.

[3] Ferraguti M, Martinez-de la Puente J, Roiz D, Ruiz S, Soriguer R, Figuerola J (2016). Effects of landscape anthropization on mosquito community composition and abundance. Sci Rep.

[4] Wilke ABB, Vasquez C, Carvajal A, Moreno M, Fuller DO, Cardenas G (2021). Urbanization favors the proliferation of *Aedes aegypti* and *Culex quinquefasciatus* in urban areas of Miami-Dade County. Florida Sci Rep.

[5] Roche B, Léger L, L'Ambert G, Lacour G, Foussadier R, Besnard G (2015). The spread of *Aedes albopictus* in metropolitan France: contribution of environmental drivers and human activities and predictions for a near future. PLoS ONE.

[6] Medlock JM, Hansford KM, Versteirt V, Cull B, Kampen H, Fontenille D (2015). An entomological review of invasive mosquitoes in Europe. Bull Entomol Res.

[7] Chiesura A (2004). The role of urban parks for the sustainable city. Landsc Urban Plan.

[8] Medeiros-Sousa AR, Fernandes A, Ceretti-Junior W, Wilke ABB, Marrelli MT (2017). Mosquitoes in urban green spaces: using an island biogeographic approach to identify drivers of species richness and composition. Sci Rep.

[9] Medeiros-Sousa AR, Ceretti-Junior W, de Carvalho GC, Nardi MS, Araujo AB, Vendrami DP (2015). Diversity and abundance of mosquitoes (Diptera: Culicidae) in an urban park: larval habitats and temporal variation. Acta Trop.

[10] Zhao J, Tang T, Wang X (2020). Effects of landscape composition on mosquito population in urban green spaces. Urban Urban Green.

[11] Carlson J, Keating J, Mbogo CM, Kahindi S, Beier JC (2004). Ecological limitations on aquatic mosquito predator colonization in the urban environment. J Vector Ecol.

[12] Wilke ABB, Benelli G, Beier JC (2021). Anthropogenic changes and associated impacts on vector-borne diseases. Trends Parasitol.

[13] Vezzani D (2007). Review: artificial container-breeding mosquitoes and cemeteries: a perfect match. Trop Med Int Health.

[14] Rydzanicz K, Czulowska A, Dyczko D, Kiewra D (2021). Assessment of mosquito larvae (Diptera: Culicidae) productivity in urban cemeteries in Wroclaw (SW Poland). Int J Trop Insect Sci.

[15] Wilke ABB, Vasquez C, Carvajal A, Moreno M, Diaz Y, Belledent T (2020). Cemeteries in Miami-Dade County, Florida are important areas to be targeted in mosquito management and control efforts. PLoS ONE.

[16] Oke T (1973). City size and the urban heat island. Atmos Environ.

[17] Jenerette GD, Harlan SL, Stefanov WL, Martin CA (2011). Ecosystem services and urban heat riskscape moderation: water, green spaces, and social inequality in Phoenix, USA. Ecol Appl.

[18] LaDeau SL, Allan BF, Leisnham PT, Levy MZ (2015). The ecological foundations of transmission potential and vector-borne disease in urban landscapes. Func Ecol.

[19] Brugman VA, Hernandez-Triana LM, Medlock JM, Fooks AR, Carpenter S, Johnson N (2018). The role of *Culex pipiens* L. (Diptera: Culicidae) in virus transmission in Europe. Int J Environ Res Public Health.

[20] Vinogradova EB (2000). *Culex pipiens pipiens* mosquitoes: taxonomy, distribution, ecology, physiology, genetics, applied importance and control.

[21] Collantes F, Delacour S, Alarcon-Elbal PM, Ruiz-Arrondo I, Delgado JA, Torrell-Sorio A (2015). Review of ten-years presence of *Aedes albopictus* in Spain 2004–2014: Known distribution and public health concerns. Parasit Vectors.

[22] Li Y, Kamara F, Zhou G, Puthiyakunnon S, Li C, Liu Y (2014). Urbanization increases *Aedes albopictus* larval habitats and accelerates mosquito development and survivorship. PLoS Negl Trop Dis.

[23] Abe M, McCall PJ, Lenhart A, Villegas E, Kroeger A (2005). The Buen Pastor cemetery in Trujillo, Venezuela: measuring dengue vector output from a public area. Trop Med Int Health.

[24] Schaffner F, Kaufmann C, Hegglin D, Mathis A (2009). The invasive mosquito *Aedes japonicus* in Central Europe. Med Vet Entomol.

[25] Fouet C, Kamdem C (2019). Integrated mosquito management: is precision control a luxury or necessity?. Trends Parasitol.

[26] EUSTAT— Instituto Vasco de Estadística. Estructura general y orgánica, y calificación del suelo de la C.A. de Euskadi por ámbitos territoriales (Ha). 2022. 2023. https://www.eustat.eus/elementos/ele0005400/estructura-general-y-organica-y-calificacion-del-suelo-de-la-ca-de-euskadi-por-ambitos-territoriales-ha/tbl0005454_c.html . Accessed 8 Feb 2024.

[27] EUSTAT–Instituto Vasco de Estadística. Población de la C.A. de Euskadi por ámbitos territoriales, según sexo y densidad de población. 01/01/2023. 2023. https://www.eustat.eus/elementos/ele0011400/Poblacion_de_la_CA_de_Euskadi_por_ambitos_territoriales_segun_sexo_y_densidad_de_poblacion/tbl0011429_c.html. Accessed 8 Feb 2024.

[28] Ayuntamiento de Vitoria. Vitoria-Gasteiz obtiene el premio Global Green City Award. 2021. https://blogs.vitoria-gasteiz.org/medios/2019/09/06/vitoria-gasteiz-obtiene-el-premio-global-green-city-award/. Accessed 9 Feb 2024.

[29] Gómez MV (1998). Reflective images: the case of urban regeneration in Glasgow and Bilbao. IJURR.

[30] Euskalmet. Climatología del País Vasco. 2024. https://euskalmet.beta.euskadi.eus/s07-5853x/es/contenidos/informacion/car_latitud/es_7257/es_latitud.html. Accessed 8 Feb 2024.

[31] González MA, Cevidanes A, Goiri F, Barandika JF, García-Pérez AL (2021). Diversity and distribution of larval habitats of mosquitoes (Diptera: Culicidae) in northern Spain: from urban to natural areas. J Vector Ecol.

[32] Becker N, Petric D, Zgomba M, Boase C, Dahl C, Madon MB (2020). Mosquitoes, identification, ecology and control.

[33] Schaffner F, Angel G, Geoffroy B, Hervy JP, Rhaiem A, Brunhes J (2001). The mosquitoes of Europe. an identification and training programme.

[34] Delgado-Serra S, Viader M, Ruiz-Arrondo I, Miranda MA, Barceló C, Bueno-Marí R (2021). Molecular characterization of mosquito diversity in the Balearic Islands. J Med Entomol.

[35] Vicente JL, Sousa CA, Alten B, Caglar SS, Falcuta E, Latorre JM (2011). Genetic and phenotypic variation of the malaria vector *Anopheles atroparvus* in southern Europe. Malar J.

[36] Collins FH, Paskewitz SM (1996). A review of the use of ribosomal DNA (rDNA) to differentiate among cryptic *Anopheles* species. Insect Mol Biol.

[37] Bahnck CM, Fonseca DM (2006). Rapid assay to identify the two genetic forms of *Culex* (*Culex*) *pipiens* L. (Diptera: Culicidae) and hybrid populations. Am J Trop Med Hyg.

[38] Estrada-Franco JG, Fernández-Santos NA, Adebiyi AA, López-López MJ, Aguilar-Durán JA, Hernández-Triana LM (2020). Vertebrate-*Aedes aegypti* and *Culex quinquefasciatus* (Diptera)-arbovirus transmission networks: non-human feeding revealed by meta-barcoding and next-generation sequencing. PLoS Negl Trop Dis.

[39] González MA, Prosser SW, Hernández-Triana LM, Alarcón-Elbal PM, Goiri F, López S (2020). Avian feeding preferences of *Culex pipiens* and *Culiseta* spp. along an urban-to-wild gradient in Northern Spain. Front Ecol Evol.

[40] R Core Team. A language and environment for statistical computing. R Foundation for Statistical Computing. 2022. https://www.r-project.org/. Accessed 8 Feb 2024.

[41] O’Hara RB, Kotze DJ (2010). Do not log-transform count data. Methods Ecol Evol.

[42] Venables WN, Ripley BD, Venables WN, Ripley BD (2002). Random and mixed effects. Modern applied statistics with S.

[43] Barton K. Multi-model inference. R Package version 1.43.17. CRAN. CRAN. 2020. https://CRAN.R-project.org/package=MuMIn. Accessed 9 Feb 2024.

[44] Oksanen J, Simpson G, Blanchet FG, Kindt R, Legendre P, Minchin P et al. Vegan community ecology package version 2.6-2. The Comprehensive R Archive Network. 2022. https://cran.r-project.org/web/packages/vegan/index.html. Accessed 30 Mar 2024

[45] Manguin S, Boete C, López-Pujol J (2011). Global impact of mosquito biodiversity, human vector-borne biseases and environmental change. The importance of biological interactions in the study of biodiversity.

[46] Kache PA, Santos-Vega M, Stewart-Ibarra AM, Cook EM, Seto KC, Diuk-Wasser MA (2022). Bridging landscape ecology and urban science to respond to the rising threat of mosquito-borne diseases. Nat Ecol Evol.

[47] Krol L, Langezaal M, Budidarma L, Wassenaar D, Didaskalou EA, Trimbos K (2024). Distribution of *Culex pipiens* life stages across urban green and grey spaces in Leiden, The Netherlands. Parasit Vectors.

[48] González MA, Goiri F, Cevidanes A, Hernández-Triana LM, Barandika JF, García-Pérez AL (2023). Mosquito community composition in two major stopover aquatic ecosystems used by migratory birds in northern Spain. Med Vet Entomol.

[49] Bravo-Barriga D, Gomes B, Almeida APG, Serrano-Aguilera FJ, Perez-Martin JE, Calero-Bernal R (2017). The mosquito fauna of the western region of Spain with emphasis on ecological factors and the characterization of *Culex pipiens* forms. J Vector Ecol.

[50] Gangoso L, Aragones D, Martinez-de la Puente J, Lucientes J, Delacour-Estrella S, Estrada PR (2020). Determinants of the current and future distribution of the West Nile virus mosquito vector *Culex pipiens* in Spain. Environ Res.

[51] Angeloni G, Bertola M, Lazzaro E, Morini M, Masi G, Sinigaglia A (2023). Epidemiology, surveillance and diagnosis of Usutu virus infection in the EU/EEA, 2012 to 2021. Euro Surveill.

[52] Haba Y, McBride L (2022). Origin and status of *Culex pipiens* mosquito ecotypes. Curr Biol.

[53] Osorio HC, Ze-Ze L, Amaro F, Nunes A, Alves MJ (2014). Sympatric occurrence of *Culex pipiens* (Diptera, Culicidae) biotypes *pipiens*, *molestus* and their hybrids in Portugal, Western Europe: feeding patterns and habitat determinants. Med Vet Entomol.

[54] Martinez-de la Puente J, Ferraguti M, Ruiz S, Roiz D, Soriguer RC, Figuerola J (2016). *Culex pipiens* forms and urbanization: effects on blood feeding sources and transmission of avian *Plasmodium*. Malar J.

[55] Bueno-Marí R, Bernués-Bañeres A, Jiménez-Peydró R (2012). Updated checklist and distribution maps of mosquitoes (Diptera: Culicidae) of Spain. Eur mosq bull.

[56] Roiz D, Eritja R, Escosa R, Lucientes J, Marques E, Melero-Alcibar R (2007). A survey of mosquitoes breeding in used tires in Spain for the detection of imported potential vector species. J Vector Ecol.

[57] Osorio HC, Ze-Ze L, Alves MJ (2012). Host-feeding patterns of *Culex pipiens* and other potential mosquito vectors (Diptera: Culicidae) of West Nile virus (Flaviviridae) collected in Portugal. J Med Entomol.

[58] Cevidanes A, Goiri F, Barandika JF, Vazquez P, Goikolea J, Zuazo A (2023). Invasive *Aedes* mosquitoes in an urban-peri-urban gradient in northern Spain: evidence of the wide distribution of *Aedes japonicus*. Parasit Vectors.

[59] Muller GC, Kravchenko VD, Junnila A, Schlein Y (2012). Tree-hole breeding mosquitoes in Israel. J Vector Ecol.

[60] Hawkes F, Medlock J, Vaux A, Cheke R, Gibson G (2020). Wetland mosquito survey handbook.

[61] Medlock JM, Vaux AG (2015). Impacts of the creation, expansion and management of English wetlands on mosquito presence and abundance - developing strategies for future disease mitigation. Parasit Vectors.

[62] Roiz D, Ruiz S, Soriguer R, Figuerola J (2015). Landscape effects on the presence, abundance and diversity of mosquitoes in Mediterranean wetlands. PLoS ONE.

[63] Koban MB, Kampen H, Scheuch DE, Frueh L, Kuhlisch C, Janssen N (2019). The Asian bush mosquito *Aedes japonicus japonicus* (Diptera: Culicidae) in Europe, 17 years after its first detection, with a focus on monitoring methods. Parasit Vectors.

[64] Hohmeister N, Werner D, Kampen H (2021). The invasive Korean bush mosquito *Aedes koreicus* (Diptera: Culicidae) in Germany as of 2020. Parasit Vectors.

[65] Kampen H, Kuhlisch C, Frohlich A, Scheuch DE, Walther D (2016). Occurrence and spread of the invasive Asian bush mosquito *Aedes japonicus japonicus* (Diptera: Culicidae) in West and North Germany since detection in 2012 and 2013, respectively. PLoS ONE.

[66] Goiri F, González MA, Goikolea J, Oribe M, Castro V, Delacour S (2020). Progressive invasion of *Aedes albopictus* in Northern Spain in the period 2013–2018 and a possible association with the increase in insect bites. Int J Environ Res Public Health.

[67] González MA, Delacour-Estrella S, Bengoa M, Barceló C, Bueno-Marí R, Eritja R (2022). A survey on native and invasive mosquitoes and other biting dipterans in northern Spain. Acta Parasitol.

[68] Robert V, Gunay F, Le Goff G, Bousses P, Sulesco T, Khalin A (2019). Distribution chart for Euro-Mediterranean mosquitoes (western Palaearctic region). J Eur Mosq Control Assoc.

[69] Giunti G, Becker N, Benelli G (2023). Invasive mosquito vectors in Europe: from bioecology to surveillance and management. Acta Trop.

[70] Mancini G, Montarsi F, Calzolari M, Capelli G, Dottori M, Ravagnan S (2017). Mosquito species involved in the circulation of West Nile and Usutu viruses in Italy. Vet Ital.

[71] Soto A, Delang L (2023). *Culex modestus*: the overlooked mosquito vector. Parasit Vectors.

[72] Vilibic-Cavlek T, Savic V, Petrovic T, Toplak I, Barbic L, Petric D (2019). Emerging trends in the epidemiology of West Nile and Usutu virus infections in southern Europe. Front Vet Sci.

[73] Soto A, De CL, Devlies AS, Van De Wiele C, Rosales Rosas AL, Wang L (2023). Belgian *Culex pipiens pipiens* are competent vectors for West Nile virus while *Culex modestus* are competent vectors for Usutu virus. PLoS Negl Trop Dis.

[74] Vogels CBF, Goertz GP, Pijlman GP, Koenraadt CJM (2017). Vector competence of northern and southern European *Culex pipiens pipiens* mosquitoes for West Nile virus across a gradient of temperatures. Med Vet Entomol.

[75] Anderson JF, Main AJ, Ferrandino FJ (2020). Horizontal and vertical transmission of West Nile virus by *Aedes vexans* (Diptera: Culicidae). J Med Entomol.

[76] Tiawsirisup S, Kinley JR, Tucker BJ, Evans RB, Rowley WA, Platt KB (2008). Vector competence of *Aedes vexans* (Diptera: Culicidae) for West Nile virus and potential as an enzootic vector. J Med Entomol.

[77] Bertola M, Mazzucato M, Pombi M, Montarsi F (2022). Updated occurrence and bionomics of potential malaria vectors in Europe: a systematic review (2000–2021). Parasit Vectors.

[78] Bueno-Mari R, Peydró R (2011). *Anopheles plumbeus* Stephens, 1828: a neglected malaria vector in Europe. Malar Rep.

[79] Schaffner F, Thiery I, Kaufmann C, Zettor A, Lengeler C, Mathis A (2012). *Anopheles plumbeus* (Diptera: Culicidae) in Europe: a mere nuisance mosquito or potential malaria vector?. Malar J.

[80] Yan J, Gangoso L, Ruiz S, Soriguer R, Figuerola J, Martinez-de la Puente J (2021). Understanding host utilization by mosquitoes: determinants, challenges and future directions. Biol Rev Camb Philos Soc.

[81] Montgomery MJ, Thiemann T, Macedo P, Brown DA, Scott TW (2011). Blood-feeding patterns of the *Culex pipiens* complex in Sacramento and Yolo Counties, California. J Med Ent.

[82] Muñoz J, Eritja R, Alcaide M, Montalvo T, Soriguer RC, Figuerola J (2011). Host-feeding patterns of native *Culex pipiens* and invasive *Aedes albopictus* mosquitoes (Diptera: Culicidae) in urban zones from Barcelona, Spain. J Med Entomol.

[83] Martinez-de la Puente J, Soriguer R, Senar JC, Figuerola J, Bueno-Mari R, Montalvo T (2020). Mosquitoes in an urban zoo: identification of blood meals, flight distances of engorged females, and avian malaria infections. Front Vet Sci.

