# Supplementary material for: Mosquitoes in urban green spaces and cemeteries in northern Spain
Source: Parasit Vectors. 2024 Apr 2;17:168. doi: 10.1186/s13071-024-06263-z (PMC10986117; doi:10.1186/s13071-024-06263-z)
Supplement: Supplementary file 1 — Additional file 1: Table S1. Commonly encountered potential hosts identified during field visits to green areas. [file 13071_2024_6263_MOESM1_ESM.docx]

**Additional file 1: Table S1.** Commonly encountered potential hosts identified during field visits to green areas.

| **City** | **Green areas** |
| --- | --- |
|  |  |
| Inland city | European rabbit (*Oryctolagus cuniculus*) |
|  | Gardeners and walkers (*Hommo sapiens*) |
|  | Diverse forest Passeriformes (*Erithacus rubecula, Parus major, Cyanistes caeruleus, Turdus merula, Pica pica*) |
|  | Eurasian hoopoe *(Upupa epops)* and common wood pigeon *(Columba palumbus)* |
| Estuarine city | Swan (*Cygnus* sp.) |
|  | Peafowl (*Pavus cristatus*) |
|  | Gardeners and walkers (*Hommo sapiens*) |
|  | Diverse Anatidae |
|  | Common pigeon (*Columba livia*) |
| Coastal city | Swan (*Cygnus* sp.) |
|  | Peafowl (*Pavus cristatus*) |
|  | Gardeners and walkers (*Hommo sapiens*) |
|  | Diverse Anatidae |
|  | Diverse forest Passeriformes: *Turdus philomelos* and *Erithacus rubecula*  Common pigeon (*Columba livia*) |
