# Supplementary material for: Mosquitoes in urban green spaces and cemeteries in northern Spain
Source: Parasit Vectors. 2024 Apr 2;17:168. doi: 10.1186/s13071-024-06263-z (PMC10986117; doi:10.1186/s13071-024-06263-z)
Supplement: Supplementary file 2 — Additional file 2: Table S2. Number of containers inspected, filled with water and with presence of larvae, by city and month. [file 13071_2024_6263_MOESM2_ESM.docx]

**Additional file 2: Table S2.** Number of containers inspected, filled with water and with presence of larvae, by city and month.

| **Parameters** | **City** | **Month** | | | | |  |
| --- | --- | --- | --- | --- | --- | --- | --- |
|  |  | **May** | **June** | **July** | **August** | **September** | **October** |
| nº of containers inspected | Inland city | 16 | 12 | 9 | 7 | 6 | 5 |
|  | Estuarine city | 25 | 25 | 20 | 9 | 9 | 6 |
|  | Coastal city | 62 | 42 | 58 | 41 | 26 | 112 |
| nº of containers with water | Inland city | 9 | 7 | 0 | 3 | 3 | 3 |
|  | Estuarine city | 15 | 14 | 6 | 5 | 7 | 2 |
|  | Coastal city | 38 | 29 | 26 | 37 | 21 | 78 |
| nº of containers with larvae | Inland city | 0 | 0 | 0 | 0 | 0 | 0 |
|  | Estuarine city | 0 | 0 | 0 | 1 | 2 | 0 |
|  | Coastal city | 0 | 4 | 6 | 5 | 5 | 7 |
